# The American fentanyl epidemic: geographic variation in mortality and policy implications

**DOI:** 10.1093/haschl/qxaf124

**Published:** 2025-06-25

**Authors:** Thomas M Wickizer, Evan V Goldstein, Rachel Mason, Nasser Sharareh

**Affiliations:** College of Public Health, The Ohio State University, Columbus, OH 43210, United States; Department of Population Health Sciences, School of Medicine, University of Utah, Salt Lake City, UT 84112, United States; College of Public Health, The Ohio State University, Columbus, OH 43210, United States; Department of Population Health Sciences, School of Medicine, University of Utah, Salt Lake City, UT 84112, United States

**Keywords:** fentanyl epidemic, fentanyl overdose, opioid mortality, opioid use disorder

## Abstract

**Introduction:**

The American fentanyl epidemic has become the worst man-made epidemic the country has faced to date, claiming tens of thousands of lives each year.

**Methods:**

Using population-based data provided by the Centers for Disease Control and Prevention, we examined the increase in unintentional, fatal fentanyl overdose since 2005 and analyzed the geographic variation in fentanyl mortality among census divisions, states, and counties.

**Results:**

In 2022, 70 813 persons died of an unintentional fentanyl overdose, a 31-fold increase over the 2139 deaths that occurred in 2012; the age-adjusted mortality rate increased similarly. Fentanyl deaths resulted in ∼2.0-2.6 million estimated years of life lost. We estimated the economic loss to the nation resulting from premature mortality was on the order of $57-$67 billion. The impact of the fentanyl epidemic varied widely by geographic area. The mortality rate of West Virginia was 15 times greater than that of South Dakota.

**Conclusion:**

Containing the fentanyl epidemic will require new, data-driven preventive and treatment approaches, coordinated across sectors, including public health, health care, law enforcement, education, and social services. Interventions should be based upon the risk profile of geographic areas and include harm reduction activities as well as social marketing campaigns to improve public awareness of fentanyl's health risks.

## Introduction

The American fentanyl epidemic has become the worst man-made epidemic the United States has faced to date, claiming tens of thousands of lives each year. Unintentional overdose deaths due to fentanyl accounted for 70 891 deaths in 2022.^1^ As we report in this paper, over the period 2005 to 2022, the United States experienced a 30-fold increase in the age-adjusted fentanyl mortality rate. First synthesized in 1960 in Belgium, fentanyl is now the opioid most widely used intravenously for intraoperative analgesia.^2,3^ Fentanyl is 50-100 times more potent than morphine, but some fentanyl analogs (carfentanyl) are many times more potent.^4,5^ Diversion of fentanyl used for medical purposes is not the cause of the fentanyl epidemic; rather, it is illegally manufactured fentanyl (IMF) imported into the United States.^6-8^

Illegally manufactured fentanyl began to appear in the United States in the late 1970s and was known as “China White.” Around 2013 large amounts of fentanyl and its chemical precursors began to be shipped to the United States from China.^9^ Later around 2019 the supply pivoted to Mexico, and it became the primary source for IMF produced by drug cartels.^9^ Because of its properties, fentanyl is relatively easy and inexpensive to produce, smuggle, and distribute.^10^ Reuter et al.^10^ estimated the cost of a morphine equivalent dose of IMF at ∼1% of heroin. The relatively inexpensive cost of IMF has been an important factor in the increased use of fentanyl.

Corresponding with the increased illegal importation of IMF came increases in fentanyl poisonings, overdoses, and deaths. Palamar et al.^11^ analyzed National Poison Control data from 2015 to 2021. The study found the proportion of the 15 391 poisonings analyzed involving fentanyl “abuse” increased by 64% from 2015 to 2021, while that involving fentanyl inhalation increased by 428%. At the same time, the proportion of major adverse events from poisoning more than doubled, increasing from 16% to 40%.^11^ The age-adjusted death rate from synthetic opioids excluding methadone was stable from 2002 (0.4 deaths per 100 000) to 2013 and then increased substantially to 22.7 in 2022.^12^

Death rates are important and widely used as an outcome measure of disease and illness. But they convey an incomplete picture of the impact of disease, especially when death occurs predominantly among younger and middle-aged adults. To provide a fuller picture of the fentanyl epidemic's impact, we report estimates of years of life lost (YLL) to unintentional, fatal fentanyl overdose. We supplemented our YLL estimates with selected estimates of the economic loss associated with fentanyl overdose. Estimating the societal economic loss that occurs with premature death, especially deaths due to fentanyl, requires assumptions that are difficult to fully justify. Despite the inherent empirical uncertainty, we believe reporting selected estimates of economic loss does have value for policy and resource allocation purposes.

We focus our analysis on fentanyl rather than opioid use disorder more generally for several reasons. First, fentanyl accounts for a great proportion of all opioid fatal overdoses. Second, because of its pharmacological properties, fentanyl poses much greater health risks than misuse of prescription opioids or use of illicit opioids such as heroin. Third, relative to other substances, the high lethality and rapid onset of action of fentanyl often preclude life-saving intervention efforts when it has been used. Fourth, public health interventions focused specifically on fentanyl are likely to be more effective than interventions more broadly focused on opioid use disorder.

Prior analyses have used measures such as YLL and economic loss (or burden) to examine the impact of substance abuse and opioid use disorder. Wickizer^13^ found substantial increases in Washington State from 1996 to 2005 in YLL and the economic costs associated with substance abuse. The research findings helped policymakers argue for, and receive, increased funding for substance abuse treatment and prevention. Gomes et al.^14^ examined the burden of opioid-related mortality from 2001 to 2016 in the United States using YLL. Other studies have assessed the economic burden of opioid use disorder and fatal opioid overdose but have not reported data specific to fentanyl.^15,16^

One recent study examined YLL related to opioid and fentanyl overdose in Ohio and found the proportion of opioid overdose due to fentanyl increased from 7.5% to 69% from 2010 to 2017.^17^ Another study by Florence et al.^18^ at the Centers for Disease Control and Prevention (CDC) examined the national economic burden of opioid use disorder and opioid fatal overdose for 2017. That study reported economic costs for health care, substance abuse treatment, criminal justice, lost productivity, and the value of life lost.

Our study was guided by 3 aims: (1) to describe trends in fentanyl-related death rates from 2005 to 2022; (2) to analyze the number of deaths and mortality rates due to unintentional fentanyl overdose for 2022 and the estimated YLL resulting from premature death; and (3) to examine the variation in mortality rates and estimated YLL among census divisions, states, and counties. A fourth aim, of secondary importance, was to examine the economic loss due to fentanyl overdose deaths.

## Data and methods

We used multiple cause of death data available through the CDC’s WONDER system.^1^ Data extracted from the interactive WONDER database are based on death certificates for US residents. Each death certificate contains a single underlying cause of death, up to 20 additional multiple causes, and demographic data. Although decedent-level data are not available through the WONDER system, we were able to obtain summary data on the number of deaths and death rates by place of residence (United States, state, and county).

For our fourth aim, we used additional data from the US Social Security Administration's Actuarial Life Tables,^19^ World Bank's National Accounts Files,^20^ US Bureau of Economic Analysis’ Regional Accounts Tables,^21^ and Federal Reserve Economic Data System's Labor Force Participation Rate Tables,^22^ as described in the Appendix.

### Data analysis

We examined unintentional deaths associated with fentanyl in the United States. To identify unintentional fentanyl-related deaths in the WONDER system, we extracted data on decedents with underlying causes of death associated with unintentional drug-/alcohol-induced poisonings (ICD-10 codes X40-X44) and the T40.4 multiple cause of death ICD-10 code. The ICD-10 code T40.4 represents deaths associated with “other synthetic narcotics,” almost all of which are related to fentanyl and fentanyl analogs.^23,24^

We first examined the age-adjusted fentanyl death rate per 100 000 persons in the United States from 2005 to 2022. The age-adjusted death rates were calculated by applying the age-specific rates of various populations to a single standard population—the 2000 US standard population. We then examined the number of deaths within age categories by sex and the (crude) death rates within the age categories.

In addition, we estimated the YLL for each age–sex category. The Actuarial Life Tables provided the estimated years of remaining life by sex and age. We calculated the average estimated years of life remaining for decedents in each age-at-death group by sex. To estimate the YLL due to fentanyl for all decedents in each age-at-death group, we multiplied the number of deaths in each age-at-death group by the average of the estimated years of life remaining for decedents in that age-at-death group. Our methods for deriving state-level YYL estimates are described in the Appendix.

We report 2 YLL estimates. The first estimate is based on an unrestricted counterfactual that assumes fentanyl decedents would have had a life expectancy equal to the population average life expectancy for their age–sex group, based upon the US Social Security Administration's Actuarial Life Tables. The second estimate restricts this counterfactual and assumes fentanyl decedents would have had a 25% shorter life expectancy due to various risks, such as overdose from other substances, chronic disease, accidents, socioeconomic factors, and physiological changes. This assumption is based on previous research demonstrating a reduction in life expectancy by about 25% among men and women with histories of opioid dependence who were receiving treatment.^25^ Our method of estimating YLL follows the same general method used by researchers at the University of Washington Institute for Health Metrics and Evaluation in their well-known studies of the global burden of disease.^26^

We conducted additional descriptive analyses to document geographic variation in fentanyl deaths, mortality rates and YLL for census divisions, states, and counties. We first examined the number of deaths and mortality rates for the 9 census divisions, representing geographic regions of different sizes and populations. Our state-level analysis compares mortality measures for the 10 states with the highest fentanyl mortality rates and the 10 states with the lowest rates among the states with available data.

To illustrate the geographic variation of fentanyl-related deaths by county for the United States, we used ArcGIS Pro to map fentanyl-related death rates by US county. We combined data from 2021 and 2022 to increase the number of counties that were not suppressed by the CDC. We organized the death rates into quartiles using ArcGIS’ quantile method, which assigns the same number of data values to each of 4 classes. To illustrate the map, we used the “North America Albers Equal Area Conic” map projection as it poses minimal distortion and preserves the area.

As a supplemental analysis to accomplish our fourth aim, we estimated the indirect societal economic loss resulting from premature death due to fentanyl for the nation overall and for selected states following a human capital approach used by other authors.^27,28^ A detailed description of this analysis is provided in the Appendix.

The Ohio State University Institutional Review Board determined that the study was not human subject research and required no further review.

## Results

### Fentanyl impact measures: US overall

**Figure 1.**
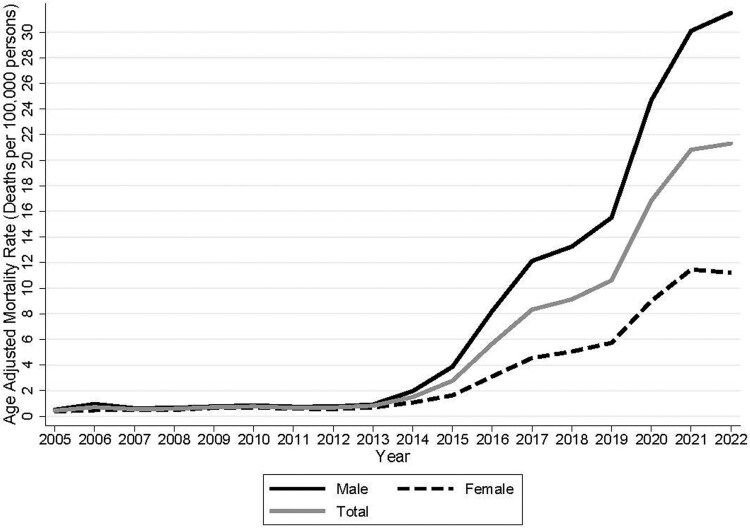
Authors’ analysis of data from the Centers for Disease Control and Prevention's WONDER online database.

Figure 1 shows the change in annual, age-adjusted fentanyl mortality rate for the period 2005-2022. As shown, there was little change in the mortality rate from 2005 to 2013. In 2013, the total fentanyl mortality rate was 0.82 per 100 000. Starting in 2013, when increased IMF was imported into the United States from China, the mortality rate began climbing, particularly for males. By 2022, the male fentanyl mortality rate increased to 30.1 per 100 000 and the total mortality rate rose to 20.82. As Figure 1 shows, much of the increase occurred after 2019 when Mexico became the dominant IMF production site and began exporting large quantities of IMF into the United States.^9^

Table 1 presents national data showing number of deaths, mortality rate, and YLL for persons aged 5 to 84 dying of unintentional fentanyl overdose in 2022. The calculation of the estimated YLL shown in the table required the data to be grouped by 10-year, age-at-death categories starting at age 5 years through 84 years. Thus, Table 1 excludes a small number of fentanyl decedents aged <5 and >84 years (*n* = 78) included in Figure 1 (*n* = 70 891).

**Table 1.** Fentanyl mortality, years of life lost by age, 2022.

	Crude death rate (per 100 000)	Deaths	Years of life lost—*Unrestricted counterfactual*	Years of life lost—*Restricted counterfactual*
**Male**				
5-14 years	0.2	40	2608	1956
15-24 years	16.0	3628	201 354	151 016
25-34 years	56.4	13 069	605 095	453 821
35-44 years	64.8	14 344	536 466	402 349
45-54 years	50.1	10 151	292 349	219 262
55-64 years	40.6	8401	175 581	131 686
65-74 years	14.1	2244	31 192	23 394
75-84 years	1.8	136	1102	826
**Female**				
5-14 years	0.2	31	2195	1646
15-24 years	7.3	1585	96 527	72 395
25-34 years	20.6	4601	236 031	177 023
35-44 years	24.3	5238	219 472	164 604
45-54 years	18.3	3680	120 336	90 252
55-64 years	13.8	2964	71 432	53 574
65-74 years	3.7	670	10 854	8141
75-84 years	0.3	31	295	221
Total		70 813	2 602 887	1 952 165

Authors’ analysis of data from the Centers for Disease Control and Prevention's WONDER Online Database and Social Security Administration's Actuarial Life Tables. The *unrestricted counterfactual* assumes fentanyl decedents would have had a life expectancy equal to the population average life expectancy for their age–sex group. The *restricted counterfactual* assumes fentanyl decedents would have had a 25% shorter life expectancy.

In 2022, 18 800 females and 52 013 males aged 5-84 years died from fentanyl overdose. Males and females aged 25-44 years accounted for over half (53.6%) of the 70 813 fentanyl deaths. The deaths given in Table 1 resulted in ∼2.0-2.6 million YLL depending upon the assumed life expectancy. Deaths among persons aged 25-44 years accounted for ∼61% of the YLL.

### Variation in fentanyl impact measures by geographic area

We began our analysis by examining the variation in fentanyl deaths and mortality rates among the 9 US Census Divisions. We observed large variations in the impact of the fentanyl epidemic among the census divisions. The fentanyl mortality rate ranged from 11.6 per 10 000 in the West South Central Division to 34.9 in New England. The 3 census divisions with the greatest number (39 066) of fentanyl deaths, Middle Atlantic, East North Central, and South Atlantic Divisions, accounted for 55% of the 70 891 fentanyl deaths occurring in 2022. Two census divisions, South Atlantic and East North Central Divisions, accounted for 40% of all YLL in the United States, resulting from fatal fentanyl overdose. The divisions with the lowest death rates included the West North and South Central Divisions and the Mountain and Pacific Divisions. The death rates among these 4 divisions ranged from 11.6 to 18.7 per 100 000.

Table 2 shows comparative data for deaths and mortality rates for the 10 states with the highest mortality rates and the 10 states with the lowest rates. The differences paint a stark contrast of the fentanyl epidemic's impact on different states. West Virginia had the highest mortality rate at 74.69 (per 100 000) followed by the District of Columbia (58.33). In contrast, the mortality rates of South Dakota and Hawaii were, respectively, 5.03 and 6.19 (per 100 000). Among the states given in Table 2, Ohio had the greatest number of deaths (3917) followed by North Carolina (3317) and Tennessee (2789). Although Texas had a moderately low mortality rate (10.35 per 100 000), it had the fourth largest number of deaths (2386) due to its large population.

**Table 2.** States with highest and lowest fentanyl mortality for persons aged 16-84 by crude death rate, 2022.

High mortality states (N = 10)	Deaths	Crude death rate (per 100 000)
West Virginia	1066	74.69
District of Columbia	319	58.33
Delaware	434	53.27
Tennessee	2789	50.03
Maine	560	49.34
Kentucky	1580	44.55
Connecticut	1240	42.64
Ohio	3917	42.31
Vermont	220	41.32
North Carolina	3317	39.06

Low mortality states (N = 10)	Deaths	Crude death rate (per 100 000)
Arkansas	306	12.90
Idaho	192	12.79
Wyoming	54	11.82
Montana	98	10.96
Texas	2386	10.35
Iowa	193	7.76
Utah	194	7.69
Nebraska	98	6.51
Hawaii	70	6.19
South Dakota	35	5.03

**Source:** Authors’ analysis of data from the Centers for Disease Control and Prevention's WONDER Online Database.

Consistent with the data given in Table 2, we found large differences in the YLL due to fentanyl. Due to limitations of state-level data (age–sex categories are unavailable), we do not report YLL estimates for each state listed in Table 2. But it is useful to make selected, limited comparisons. Using the more conservative, restricted counterfactual assumption that lowered life expectancy by 25%, we estimated West Virginia, Delaware, and Washington DC, respectively, would have had an estimated 25 185, 10 254, and 7537 YLL. States included in Table 2 with low death rates incurred far fewer YLL. Nebraska, Hawaii, and South Dakota would have had an estimated 2316, 1654, and 828 YLL, respectively. These data clearly show the impact of the fentanyl epidemic measured in YLL varied substantially among states.

**Figure 2.**
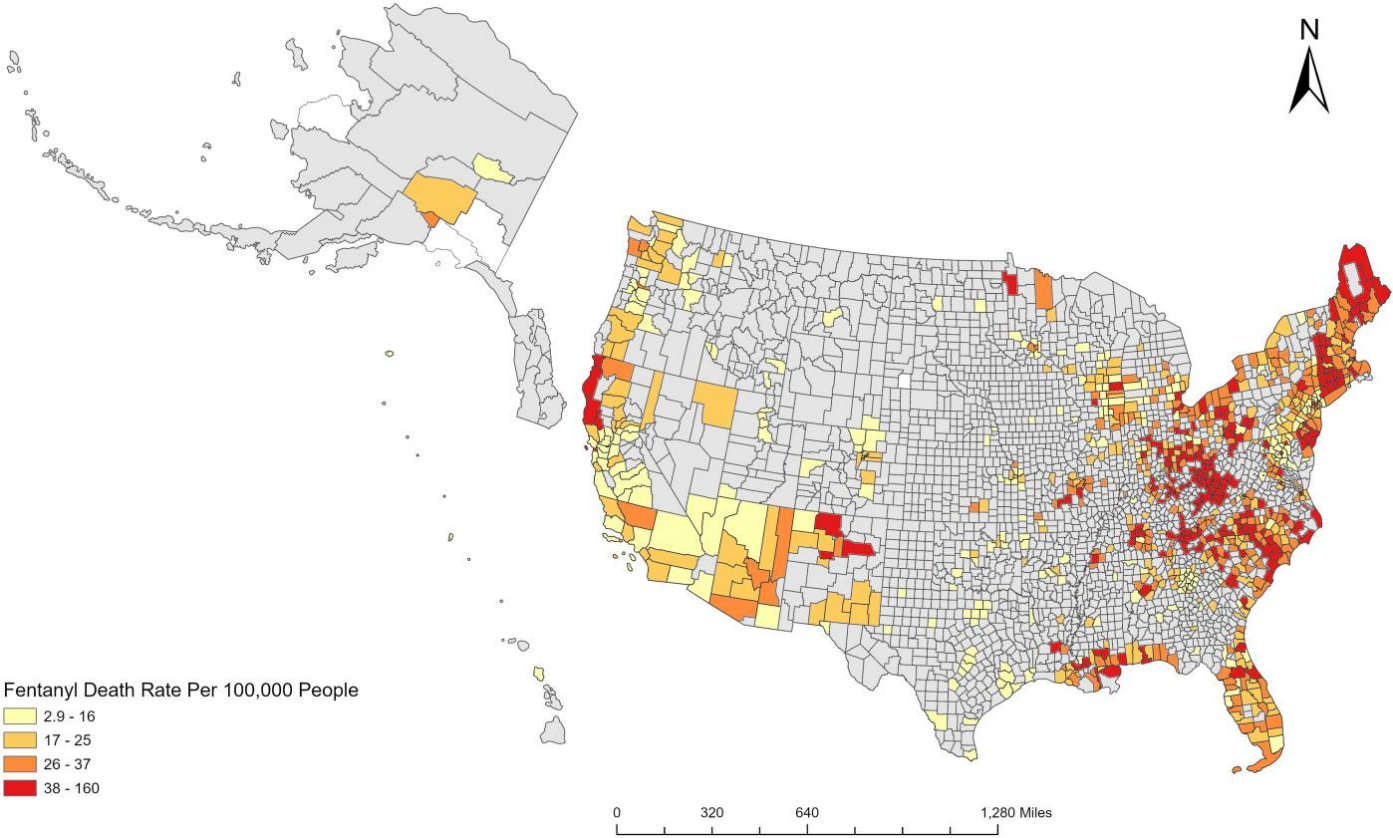
Authors’ analysis of data from the Centers for Disease Control and Prevention's WONDER online database. ArcGIS Pro was used to map the geographic variation of fentanyl-related deaths by county for the United States. Data from 2021 and 2022 were combined to increase the number of counties that were not suppressed by the CDC. The death rates shown were organized into quartiles using ArcGIS’ quantile method, which assigns the same number of data values to each of 4 classes.

Figure 2 depicts the death rates by quartile for 927 counties out of 3142 counties that had reportable mortality data. As shown, there is clear clustering of counties representing the top quartile of death rates (38.0-160.0 per 100 000). These counties are mostly located in New England, as well as the South Atlantic and the East South Central Census Divisions and Ohio. But high death rate counties appear elsewhere, in particular northwestern California, New Mexico, and Louisiana. Counties representing the second quartile (26.0-37.0 deaths per 100 000) also cluster in the same census divisions and states as those in the top quartile. The 2215 counties that had too few fentanyl deaths (*n* ≤ 10) to be reportable in 2021 and 2022 are located predominantly in the Midwest and Pacific Census Divisions.

### Economic loss associated with fentanyl mortality

The impact of a disease or illness resulting in premature death can be considered as a societal economic loss. Society suffers an economic loss when people die prematurely because they no longer produce goods or services, consume products, or pay taxes. Table 1 shows the majority of persons dying from fentanyl overdose were young or middle-aged adults 25-44 years. Readers should understand considerable uncertainty exists in estimating the economic loss associated with premature death due to fentanyl. Accordingly, we present a range of economic loss estimates based on assumptions of different degrees of restrictiveness. Our lower-bound estimate assumes fentanyl decedents would have had a 25% lower life expectancy than that of the population average. This assumption yields a national economic loss, measured in net present value, on the order of $57.0 billion due to the 70 813 premature fentanyl deaths recorded in 2022 (Appendix Table S1). Relaxing this assumption and instead assuming population average life expectancy, yields an upper-bound estimate of economic loss on the order of $67 billion. Decedents aged 25 to 44 years accounted for 60% of the total economic loss.

States play a prominent role in allocating resources for prevention and treatment to combat the fentanyl epidemic. Consistent with the state-level differences we showed for YLL, we found large differences in economic losses due to fentanyl among states. Ohio incurred the largest estimated economic loss due to fentanyl mortality among the states given in Table 2. Using the more conservative assumption regarding life expectancy, we estimated Ohio would have incurred a loss of $3.0 billion resulting from the 3917 fentanyl deaths that occurred in 2022 (data not shown). North Carolina suffered the second greatest economic loss estimated at $2.4 billion. In contrast, states that had far fewer deaths and lower mortality rates suffered considerably smaller economic losses. South Dakota, with the lowest death rate of 5.03 per 100 000, incurred an estimated economic loss of $26.7 million. Nebraska, with a death rate of 6.51 per 100 000, had an estimated economic loss of $95.8 million.

### Limitations

Our analysis has several limitations worth noting. Ascertainment of death by fentanyl as reported on death certificates involves inherent uncertainty, probably leading to some unknown degree of undercounting. The certifier signing the death certificate (physician, medical examiner, or coroner) may miscode the cause of death, a problem likely to occur with greater frequency for fentanyl overdose.^29^ While our analysis accounted for labor force participation, a strength of our study, it made the implicit assumption fentanyl deaths occurred in persons whose labor force participation was representative of the general US population. Due to data limitations, in particular lack of data for age–sex groups, our state-level estimates of YLL and economic loss have greater uncertainty than our national estimates.

Our supplemental economic cost analysis did not consider other costs related to morbidity, healthcare, or law enforcement arising from unintentional fentanyl use or misuse. In general, readers should understand various methods and considerable uncertainty exist in estimating the economic loss associated with premature death due to various causes. Finally, the WONDER system does not provide decedent-level data. Instead, the system provides data aggregated by demographic and geographic categories. Our analyses used fentanyl-related death data for decedents of various age ranges. Thus, we could not account for the exact distribution of deaths or life expectancies within each group.

## Discussion

The American fentanyl epidemic, currently the number 1 cause of death in the United States for persons aged 18-45 years, has continued to claim more lives every year, although the number of unintentional deaths from fentanyl now appears to be decreasing based on CDC provisional 2024 mortality data. The fentanyl mortality rate rose sharply between 2019 and 2022, increasing from ∼10.6-21.7 per 100 000. In 2022, 70 813 persons aged 5-84 years died from unintentional fatal fentanyl overdose, resulting in ∼2.0-2.6 million YLL and an associated economic loss on the order of $57-$67 billion. Persons aged 25-44 years accounted for over half (52.6%) of the deaths and for 61.4% of the estimated YLL.

The impact of the fentanyl epidemic varied substantially by geographic area. The mortality rate ranged from 11.56 per 100 000 in the West South Central Division to 34.93 in the New England Division. We observed similar large variations in mortality by state, with South Dakota and Hawaii having the lowest mortality rates, 5.03 and 6.19 per 100 000, and West Virginia and the District of Columbia having the highest rates, 74.69 and 58.33 per 100 000.

In our analysis, the fentanyl epidemic claimed the most lives in 2022 in Ohio (3917) followed by North Carolina and Tennessee, 3317 and 2789, respectively. Consistent with deaths and mortality rates, the estimated economic loss varied widely by state. Among the states given in Table 2, Ohio incurred the greatest economic loss estimated conservatively at ∼$3.0 billion. In contrast, South Dakota and Hawaii, with fewer than 75 total fentanyl deaths each, incurred a combined economic loss of less than $84 million.

## Conclusion

The findings reported here for 2022 have documented the number of deaths, mortality rates, estimated YLL, and economic costs associated with unintentional, fatal fentanyl overdose. We found the impact of the fentanyl epidemic varied widely across regions and states as well as local counties. Thus, though often thought of as a national epidemic, the fentanyl epidemic is better characterized as a local, state, or regional epidemic. As such, our findings have important policy implications for both prevention and treatment. Specifically, data-driven approaches that incorporate epidemiologic risk assessments at the local, state, and regional levels are needed to address the fentanyl epidemic occurring across various American communities. Regional and local prevention efforts are particularly crucial given potential disinvestments in public health programs at the federal level.

Public health interventions should be developed based on the risk profile of specific geographic areas. Such interventions will require organizational leadership and coordination across sectors, including public health, healthcare, law enforcement, education, and social services. Harm reduction activities^30,31^ as well as social marketing campaigns^32,33^ should be components of the interventions. Studies show such activities and campaigns do have potential to reduce the risk of fentanyl overdose.^34-37^

In sum, local communities, states, and regions need to become “activated” to combat the fentanyl epidemic. The concept of community activation to improve health is not new. Researchers at the Group Health Center for Health Studies (now the Kaiser Permanente Washington Health Research Institute) and the University of Washington used the concept of community activation to evaluate the implementation of a major, community health promotion and disease prevention demonstration in western states during the late 1980s.^38-40^ Now, as then, community activation is needed to create networks of well-informed local stakeholders who can share harm reduction information, implement evidence-based interventions in community settings, connect people with life-saving resources, and affect the public policy process.

In each of the demonstration communities, coalitions were formed to facilitate coordination of prevention activities among different agencies and organizations. Such coalitions could be formed at the local, state, or regional levels and play an important role in coordinating interventions among organizations combating the fentanyl epidemic. The recently completed NIH-funded HEALing Communities Study provides important lessons about the role community coalitions and engaged partnerships can play in reducing the risk of opioid overdose.^37^

Wickizer and Mason conducted an online survey in 2023 of 72 harm reduction programs located around the country to assess their organization and operations. An exemplar program that appeared to have achieved considerable success in helping to activate its community to combat the fentanyl epidemic was the program located in Cleveland, Ohio, funded by the Alcohol, Drug Addiction, and Mental Health Services (ADAMHS) Board of Cuyahoga County. The Cleveland harm reduction program coordinates with organizations in different sectors, including public health, healthcare, social services, law enforcement, and education, to promote preventive services and harm reduction activities. The ADAMHS program uses data from different organizations and sectors, including law enforcement, to develop strategies aimed at combating the fentanyl epidemic.

Increased resources will be needed to combat the fentanyl epidemic. The question for policymakers raised by our findings is: In light of the mortality, estimated YLL and economic loss due to fentanyl, are we allocating sufficient resources to combat the fentanyl epidemic? Finally, we note the estimates of YLL and economic loss presented here do not begin to capture the enormous intangible costs the fentanyl epidemic has had on American families, communities, and the nation overall.

## Supplementary Material

qxaf124_Supplementary_Data
