# Supplementary material for: The American fentanyl epidemic: geographic variation in mortality and policy implications
Source: Health Aff Sch. 2025 Jun 25;3(7):qxaf124. doi: 10.1093/haschl/qxaf124 (PMC12247507; doi:10.1093/haschl/qxaf124)
Supplement: qxaf124_Supplementary_Data [file qxaf124_supplementary_data.zip › Appendix Table S1.docx]

**Table A1. Societal Economic Loss Due to Unintentional Fentanyl Deaths, by Sex and Age, 2022**

|  | **Economic Loss (Present Value) –**  ***Unrestricted Counterfactual*** | **Economic Loss (Present Value) –**  ***Restricted Counterfactual*** |
| --- | --- | --- |
| **Male** |  |  |
| 5-14 years | $ 42,935,539 | $ 36,777,964 |
| 15-24 years | $ 4,933,295,930 | $ 4,330,325,476 |
| 25-34 years | $ 16,807,068,862 | $ 14,460,443,593 |
| 35-44 years | $ 15,477,538,344 | $ 13,054,434,852 |
| 45-54 years | $ 8,303,785,918 | $ 6,836,584,250 |
| 55-64 years | $ 4,334,114,038 | $ 3,491,312,138 |
| 65-74 years | $ 452,286,923 | $ 346,577,970 |
| 75-84 years | $ 8,839,827 | $ 6,849,423 |
| **Female** |  |  |
| 5-14 years | $ 30,138,616 | $ 25,936,234 |
| 15-24 years | $ 1,942,281,853 | $ 1,724,106,013 |
| 25-34 years | $ 5,299,145,368 | $ 4,613,648,391 |
| 35-44 years | $ 5,072,981,843 | $ 4,319,357,057 |
| 45-54 years | $ 2,738,141,502 | $ 2,276,981,858 |
| 55-64 years | $ 1,371,696,839 | $ 1,115,441,250 |
| 65-74 years | $ 108,875,099 | $ 84,458,904 |
| 75-84 years | $ 1,312,660 | $ 1,015,164 |
| **Total** | **$ 66,924,439,161** | **$ 56,724,250,538** |

**NOTES:** Authors’ analysis of data from the Centers for Disease Control and Prevention’s WONDER Online Database, Social Security Administration’s Actuarial Life Tables, World Bank National Accounts, US Bureau of Economic Analysis, the Federal Reserve Economic Data (FRED). The *unrestricted counterfactual* assumes fentanyl decedents would have had a life expectancy equal to the population average life expectancy for their age-sex group. The *restricted counterfactual* assumes fentanyl decedents would have had a 25% shorter life expectancy.
