# Supplementary material for: The American fentanyl epidemic: geographic variation in mortality and policy implications
Source: Health Aff Sch. 2025 Jun 25;3(7):qxaf124. doi: 10.1093/haschl/qxaf124 (PMC12247507; doi:10.1093/haschl/qxaf124)
Supplement: qxaf124_Supplementary_Data [file qxaf124_supplementary_data.zip › HAS Appendix.docx]

**Appendix**

**Societal Cost Estimate**

***Rationale***

We estimated the indirect societal cost of YLL due to unintentional death from fentanyl in the US by sex (male, female) and age-at-death group (5-14 years, 15-24 years, 25-34 years, 35-44 years, 45-54 years, 55-64 years, 65-74 years, 75-84 years) in 2022. To conduct this analysis, we followed a human capital approach used by other authors^1,2^ for a range of causes of death. All estimated economic values represent indirect costs from a societal perspective (i.e., an indirect valuation of life and productivity). The rationale for estimating indirect economic losses is that premature death depletes economic output (i.e., Gross Domestic Product, or GDP) when individuals are unable to save, invest, consume, pay taxes, or produce commodities as a person would across the typical lifespan. Readers should understand considerable uncertainty exists in estimating the economic loss associated with premature death due to fentanyl. Accordingly, we calculated a range of economic-loss estimates based on assumptions of different degrees of restrictiveness (i.e., unrestricted and restricted counterfactuals).

***Method***

At the national level, the total estimated economic value of YLL (TCYLL) of unintentional death from fentanyl was the sum of the potential GDP lost due to unintentional fentanyl deaths among males aged 5–14 years (MCYLL_5−14_), 15–24 years (MCYLL_15−24_), 25–34 years (MCYLL_25−34_), 35–44 years (MCYLL_35−44_), 45–54 years (MCYLL_45−54_), 55-64 years (MCYLL_55−64_), 65-74 years (MCYLL_65−74_), and 75–84 years (MCYLL_75−84_) and females aged 5–14 years (FCYLL_5−14_), 15–24 years (FCYLL_15−24_), 25–34 years (FCYLL_25−34_), 35–44 years (FCYLL_35−44_), 45–54 years (FCYLL_45−54_), 55-64 years (FCYLL_55−64_), 65-74 years (FCYLL_65−74_), and 75–84 years (FCYLL_75−84_), represented by *Equation 1*:

$$TCYLL = \left( MCYLL_{5-14}+ MCYLL_{15-24}+ \ldots+ FCYLL_{65-74}+ FCYLL_{75-84} \right)$$

We took the following steps to calculate these values. First, by sex, we extracted the total number of deaths attributable to fentanyl by age-at-death group – 5-14 years, 15-24 years, 25-34 years, 35-44 years, 45-54 years, 55-64 years, 65-74 years, 75-84 years – from the CDC WONDER system. Second, we estimated the YLL for each age-at-death group (e.g., YLL_z_ or YLL_z_, where *z* is the age-at-death group) using data from the Social Security Administration’s Actuarial Life Tables.^3^ The Actuarial Life Tables provided the estimated years of remaining life by sex and age, e.g., about 46.3 years for males ages 25-34 years. Using this information, assuming the unrestricted counterfactual, we multiplied the number of deaths in each age-at-death group (*z*) by the estimated years of life remaining per decedent in the age-at-death group to represent the estimated total YLL due to fentanyl for all decedents in that age-at-death group. For example, for the male 25-34 years age-at-death group, we multiplied 3,628 deaths by 46.3 years (i.e., the average remaining life expectancy for males ages 25-34 in the Actuarial Life Tables) to derive our estimate of 201,354 YLL. Comparatively, to produce our restricted counterfactual estimate, we multiplied the estimated years of remaining life for each age-at-death group by 0.75.

Third, to estimate the economic value of the YLL for each age-at-death group, we multiplied GDP per capita (current US$, 2022) by the total number of decedents in each age-at-death group for each estimated year of life remaining, discounting each year by 3% to convert future economic losses to present value (i.e., the present value of life), as represented by

*Equation 2:*

$$MCYLL_{z}/ FCYLL_{z}= \sum_{i=1}^{k} \left\{ \left[ \frac{1}{\left( 1+r \right)^{k}} \right]\times\left[ {GDP per capita}_{\$} \right]\times\left[ {Deaths}_{z} \right] \right\}= \left\{ \left[ \frac{1}{\left( 1+r \right)^{1}} \right]\times\left[ {GDP per capita}_{\$} \right]\times\left[ {Deaths}_{z} \right] \right\}+ \left\{ \left[ \frac{1}{\left( 1+r \right)^{2}} \right]\times\left[ {GDP per capita}_{\$} \right]\times\left[ {Deaths}_{z} \right] \right\}+ \ldots+ \left\{ \left[ \frac{1}{\left( 1+r \right)^{k}} \right]\times\left[ {GDP per capita}_{\$} \right]\times\left[ {Deaths}_{z} \right] \right\}$$

where MCYLL_z_ or FCYLL_z_ is the estimated economic value of YLL among persons of *z* age-at-death group for males or females, respectively; *1/(1 + r)^k^* is the discount factor used to convert future economic losses to present values; *r* is the discount rate measuring the opportunity cost of lost output (3% in the our study); $\sum_{i=1}^{k}$ is the summation for the estimated years of life remaining per age-at-death group, i.e., the data retrieved from the Social Security Administration’s Actuarial Life Tables described above; *k* is the final year of the estimated years of life remaining; and *Deaths_z_* is the total number of decedents in each age-at-death group. GDP per capita information was accessed through the World Bank’s National Accounts Files.^4^

Next, for each sex and age-at-death group, we multiplied these subtotals by the average of the labor force participation rates forecasted over the period 2022-2032, downloaded in table format by sex and age from the Bureau of Labor Statistics,^5^ assuming not all decedents would have participated in the workforce or contributed to the economy. National labor force participation rate projections were unavailable beyond 2032 at the time of this analysis. Thus, we assumed that labor force participation rates would be similar to the average of the labor force participation rates forecasted for 2022-2032 over the projected period of years of life remaining per age-at-death group.

Finally, we summed the subtotal estimates of the present value of labor-force-participation-rate-adjusted economic losses for all age-at-death groups by sex to produce our estimate of the total present economic value of YLL to fentanyl in the US in 2022 (i.e., TCYLL). The unrestricted and restricted counterfactuals followed the same steps except for the 25% reduction in estimated YLL used for the restricted counterfactual estimates (see Step #2 above).

Counts and rates of fentanyl deaths for decedents <5 and >84 years of age were suppressed and/or deemed unreliable for use by the CDC and not included in this analysis. It was also assumed that decedents were not employed until the age of 16; thus, economic values were not calculated for the 7 initial years of life expectancy for decedents in the 5-14 years age-at-death group.

***State-level Cost Estimates***

We also estimated the indirect societal cost of YLL to unintentional death from fentanyl for states with the highest and lowest fentanyl death rates in 2022. This analysis mirrored the approach described above, including estimated YLL and economic losses for both the unrestricted and restricted counterfactuals, except for two distinctions. First, only decedents ages 16-84 were included in this analysis, aligning with the states’ working-age population. This was because the WONDER system does not provide decedent-level data. Instead, the system provides data aggregated by demographic and geographic categories, but fentanyl-related death data by age-at-death group were suppressed and/or deemed unreliable for use by the CDC at the state level. Working-age population adjustments could not be made at the state level if decedents <16 years of age were included. Further, the state-level estimates were multiplied by the average of each state’s labor force participation rates from the five previous years (2018-2022), not future years, because state-level labor force participation rate projections are unavailable. Thus, for the state-level analysis, we assumed future labor force participation rates would be similar to the average of labor market conditions from previous years.^6^ Each state’s labor force participants rates from 2018-2022 and 2022 GDP per capita data were gleaned from the Federal Reserve Economic Data (FRED) System’s Labor Force Participation Rate Tables and US Bureau of Economic Analysis’ (BEA) Regional Accounts Tables, respectively.^7-8^

**References**

1. Kirigia JM, Muthuri RDK, Muthuri NG. The monetary value of human lives lost to suicide in the african continent: Beating the african war drums. Healthcare. 2020;8(2):84-91.
2. Kirigia JM, Masiye F, Kirigia DG, Akweongo P. Indirect costs associated with deaths from the Ebola virus disease in West Africa. Infect Dis Poverty. 2015;4:45-52.
3. U.S. Social Security Administration. Actuarial Life Tables. https://www.ssa.gov/oact/STATS/table4c6.html. Published 2024. Accessed May 1, 2024.
4. World Bank. GDP Per Capita Tables. https://data.worldbank.org/indicator/NY.GDP.PCAP.CD?locations=US. Published 2024. Accessed May 1, 2024.
5. U.S. Bureau of Labor Statistics. Labor Force Characteristics. <https://www.bls.gov/cps/lfcharacteristics.htm>. Published 2017. Accessed May 1, 2024.
6. Ortega-Ortega M, Hanly P, Pearce A, Soerjomataram I, Sharp L. Projected impact on labour productivity costs of cancer-related premature mortality in Europe 2018–2040. Appl Health Econ Policy. 2023;21(6):877-889.
7. Federal Reserve Economic Database. Labor Force Participation Rate Tables. cvhttps://fred.stlouisfed.org/release/tables?rid=446&eid=784070&od=2021-06-01. Published 2024. Accessed May 1, 2024.
8. U.S. Bureau of Economic Analysis. Gross Domestic Product by State, Annual 2022. https://apps.bea.gov/regional/histdata/releases/0323gdpstate/index.cfm. Published 2024. Accessed May 1, 2024.
